# Characterization and ligand identification of a membrane progesterone receptor in fungi: existence of a novel PAQR in *Sporothrix schenckii*

**DOI:** 10.1186/1471-2180-12-194

**Published:** 2012-09-07

**Authors:** Waleska Gonzalez-Velazquez, Ricardo Gonzalez-Mendez, Nuri Rodriguez-del Valle

**Affiliations:** 1 Department of Microbiology and Medical Zoology, Medical Sciences Campus, University of Puerto Rico, PO Box 365067, San Juan PR 00936-5067, USA; 2 Department of Radiological Sciences, Medical Sciences Campus, University of Puerto Rico, PO Box 365067, San Juan, PR 00936-5067, USA

## Abstract

**Background:**

Adaptive responses in fungi result from the interaction of membrane receptors and extracellular ligands. Many different classes of receptors have been described in eukaryotic cells. Recently a new family of receptors classified as belonging to the progesterone-adiponectin receptor (PAQR) family has been identified. These receptors have the seven transmembrane domains characteristic of G-protein coupled receptors, but their activity has not been associated directly to G proteins. They share sequence similarity to the eubacterial hemolysin III proteins.

**Results:**

A new receptor, SsPAQR1 (*Sporothrixschenckii*progesterone-adiponectinQ receptor1), was identified as interacting with *Sporothrix schenckii* G protein alpha subunit SSG-2 in a yeast two-hybrid assay. The receptor was identified as a member of the PAQR family. The cDNA sequence revealed a predicted ORF of 1542 bp encoding a 514 amino acids protein with a calculated molecular weight of 57.8 kDa. Protein domain analysis of SsPAQR1 showed the 7 transmembrane domains (TM) characteristic of G protein coupled receptors and the presence of the distinctive motifs that characterize PAQRs. A yeast-based assay specific for PAQRs identified progesterone as the agonist. *S. schenckii* yeast cells exposed to progesterone (0.50 mM) showed an increase in intracellular levels of 3^′^, 5^′^ cyclic adenosine monophosphate (cAMP) within the first min of incubation with the hormone. Different progesterone concentrations were tested for their effect on the growth of the fungus. Cultures incubated at 35°C did not grow at concentrations of progesterone of 0.05 mM or higher. Cultures incubated at 25°C grew at all concentrations tested (0.01 mM-0.50 mM) with growth decreasing gradually with the increase in progesterone concentration.

**Conclusion:**

This work describes a receptor associated with a G protein alpha subunit in *S. schenckii* belonging to the PAQR family. Progesterone was identified as the ligand. Exposure to progesterone increased the levels of cAMP in fungal yeast cells within the first min of incubation suggesting the connection of this receptor to the cAMP signalling pathway. Progesterone inhibited the growth of both the yeast and mycelium forms of the fungus, with the yeast form being the most affected by the hormone.

## Background

Heterotrimeric (αβγ) guanine nucleotide binding proteins (G proteins) constitute a family of regulatory GTP hydrolases associated with the cytoplasmic face of the plasma membrane [1-4]. Their activity is characterized by a cycle of GTP-binding and hydrolysis. The GTP- and GDP-bound complexes define the active and inactive states of the G proteins, respectively. The binding of specific ligands to transmembrane receptors activates the heterotrimeric G protein subunits that are responsible for the flow of information in many eukaryotic signal transduction pathways [5]. The traditional G proteins coupled receptors (GPCRs) share a characteristic topological structure of seven transmembrane domains and recognize diverse extracellular signals. The cytoplasmic C-terminal region contains the Gα binding activity.

Recently, a new class of seven transmembrane receptors has been identified in humans and other vertebrates and has been classified as belonging to the PAQR superfamily (progestin-adipoQ receptors) [6-10]). Their activity has not been directly associated to heterotrimeric G proteins but indirect evidence suggests that they might be associated to G protein alpha subunits [11,12].

The PAQR superfamily includes three classes of membrane receptors. Class I PAQRs are adiponectin receptors and include: AdipoR1 (PAQR 1), AdipoR2 (PAQR 2), PAQR 3 and PAQR 6 [13]. These receptors respond to adiponectin that is an insulin-sensitizing peptide hormone found in vertebrates [14,15]. Low serum adiponectin levels have been identified as a high risk factor for type 2 diabetes and other complications such as atherosclerosis and hepatic steatosis. Adiponectin has been reported to have a positive effect on insulin sensitivity and energy metabolism [16].

Class II PAQRs respond to progesterone and include: mPRα (PAQR 7), mPRβ (PAQR 8) and mPRγ (PAQR 5) [13]. For a long time progesterone had been observed to mediate immediate cellular effects not attributable to the classical nuclear progesterone receptors that involve mRNA and new protein synthesis [10]. With the identification of the PAQR membrane receptors for progesterone the rapid effects of this hormone, not dependent on gene transcription, can be explained [6]. The response of steroid membrane receptors can be rapid, as in the case of sperm hypermotility, or can occur over a prolonged period of time as in the case of oocyte maturation in fish [17] and amphibians [18,19].

Class III are the hemolysin III-related receptors that have the deepest evolutionary roots but whose agonists are not known, these are PAQR 10 and PAQR 11 [20] and the bacterial hemolysin III large class of proteins, expressed in many bacterial species [7]. The latter have been shown to induce cytolysis of eukaryotic cells by pore formation [21].

In *Saccharomyces cerevisiae,* the Izh genes encode membrane proteins that also belong to the ubiquitous protein family that includes hemolysin III and vertebrate membrane PAQR homologues. The Izh family (implicated in zinc homeostasis) consists of 4 different proteins: Izh1, Izh2, Izh3 and Izh4. All but the Izh1 have the 7 transmembrane domains of the PAQRs [22]. The agonist for Izh2 has been identified as osmotin, a plant defense protein that is a homologue of adiponectin [23]. Yeast mutants of the Izh proteins exhibit defects in zinc tolerance. Izh proteins have been reported to be regulated by exogenous fatty acids, suggesting a role in lipid metabolism [24]. The effects of Izh proteins on zinc homeostasis have been related either directly or indirectly to their effects on lipid metabolism [24].

The effects of steroid hormones in the development of the parasitic forms of pathogenic dimorphic fungi, drug resistance and susceptibility to infection, makes the identification of specific steroid receptors and steroid binding proteins of outmost importance in the treatment of fungal infections [reviewed in [25]. In *Paracoccidioides brasiliensis* the susceptibility to infection was observed to be dependent on gender, men being more susceptible than women, while in the case of *Coccidioides immitis*, pregnancy increases the risk of developing the disease [26]. In both of these cases, hormones were suggested as responsible for these differences. On the other hand, *in vitro* studies of the phase transition from mycelium to yeast in *P. brasiliensis* showed that the transition to the yeast form was inhibited in the presence of estrogen [25]. In *Candida albicans*, steroids were found to alter the response to antifungal drugs [25].

Nevertheless, the identification of progesterone membrane receptors in fungi has been elusive. As mentioned above, specific receptors for steroid hormones in pathogenic fungi have not been thoroughly studied and identified. Progesterone has been reported to bind to fungal membranes but the direct identification of specific progesterone receptors has not been reported until now. In *Rhizopus*, membrane ligand-binding assays suggest the presence of a progesterone receptor but that has not led to the identification of the specific receptor [27-30].

In this work we identified a homologue of the PAQR family as an interacting protein of the *S. schenckii* G protein alpha subunit, SSG2, using the yeast two-hybrid analysis. Using a yeast-based assay we determined that progesterone was the ligand of this *S. schenckii* PAQR (SsPAQR1). This assay was used because it is specific for PAQRs and was intended for the study of these receptors without the intervention of other possible progesterone binding proteins. The receptor was expressed in *S. cerevisiae* that has no other known progesterone receptor. We also report the effects of this agonist on the growth of the fungus from conidia and on the intracellular cyclic 3^′^, 5^′^ adenosine monophosphate (cAMP) levels in *S. schenckii* yeast cells at various time intervals following exposure to the hormone.

## Results

### Yeast two-hybrid screening

A yeast two-hybrid assay was done using the complete coding sequence of SSG-2 as bait and a *S. schenckii* yeast cells cDNA library. In this screening, a 483 bp insert from a blue colony growing in quadruple drop out (QDO) medium (SD/-Ade/-His/-Leu/-Trp/X-α-gal) was sequenced and found to encode the last 38 amino acid of the C-terminal residues of a protein homologous to Izh3 from *S. cerevisiae* (GenBank no. NP_013123.1).

### Sequencing of the *SsPAQR1* gene

**Figure 1:**
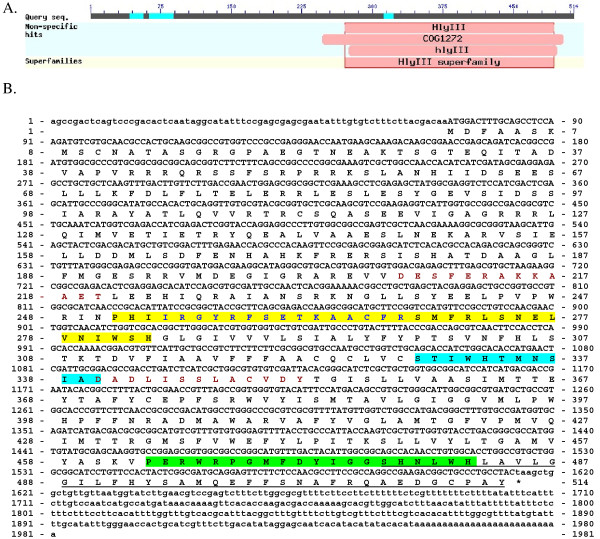
**cDNA and derived amino acid sequences of the *sspaqr1 *gene. ** Figure1A shows the hemolysin III motif identified using the NCBI Conserved Domain Database. The hemolysin III motif that is present in all PAQRs, extends from amino acid 270 to 495. Figure1B shows the cDNA and derived amino acid sequence of the *sspaqr1 * gene. Non-coding regions are given in lower case letters, coding regions and amino acids are given in upper case letters. Motif A, B and C are shaded yellow, blue-green, and green, respectively. Motif C includes part of the original sequence isolated in the yeast two-hybrid assay. The original sequence isolated using the yeast two-hybrid assay is underlined.

Figure1 shows the cDNA and derived amino acid sequence of *sspaqr1* gene obtained using 5^′^ RACE. This figure shows a 1981 bp cDNA with an ORF of 1542 bp encoding a 514 amino acid protein with a calculated molecular weight of 57.8 kDa. The GenBank accession numbers for the cDNA and derived amino acid sequence, respectively are: EU439945.1 and ACA43006.1. The PANTHER Classification System identified this protein as a member of the PAQR family (PTHR20855:SF10) (residues 149-512) with an extremely significant E value of 3.8 e^-158^[31]. 

Figure1 also shows the characteristic residues that identify the members of the Class II PAQR family of receptors. The Class II PAQR family (progesterone receptors) is characterized by the presence of 7 transmembrane domains, and three highly conserved amino acid motifs [13]. These include: motif A (shaded in yellow), consisting of the sequence Nx_3_H found N-terminal to TM1; motif B, consisting of the sequence Sx_3_H (shaded in blue-green) at the end of TM2 and an aspartic acid residue at the beginning of TM3, and motif C (shaded in green), consisting of the sequence, Hx_3_H in the loop region between TM6 and TM7 [7,13]. It is of interest to note that motif C includes part of the original sequence isolated in the yeast two-hybrid assay; this sequence is underlined in Figure1.

**Figure 2:**
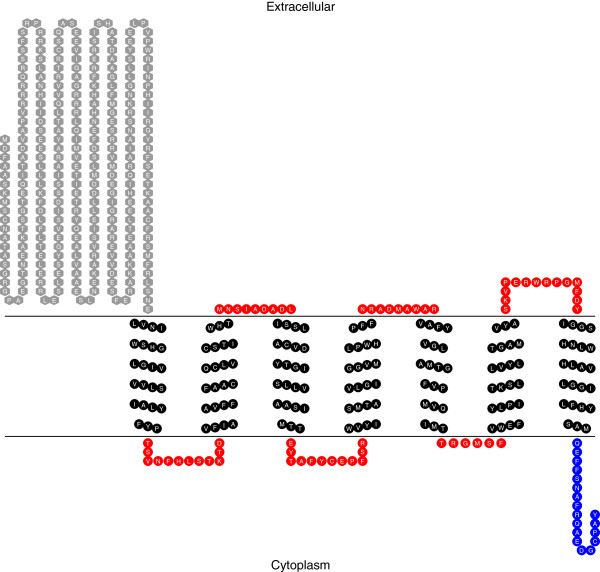
**Transmembrane domain analysis of SsPAQR1.** Figure2 shows the transmembrane domain analysis of SsPAQR1 obtained using TMHMM server v. 2.0 (http://www.cbs.dtu.dk/services/TMHMM) for the prediction of transmembrane helices. The 7 transmembrane domains that characterize this receptor family are shown. Membrane topology was visualized with TOPO2 (http://www.sacs.ucsf.edu/TOPO2/).

Figure2 shows the results obtained when the SsPAQR1 sequence was analyzed for transmembrane domains using the TMHMM server v. 2.0 and TOPO 2 [32]. This figure shows the 7 transmembrane domains that characterize these receptors. According to the TMHMM server, SOSUI server and PSIPRED Protein structure prediction server (MEMSAT-SVM) analyses [32-34], the N-terminal domain is extracellular and the C-terminal domain is intracellular as shown. PSORT II analysis identified the localization of this receptor in the plasma membrane with a 45% probability [35]. Signal peptide analysis using Predotar [36], TargetP [37] or MitoProt [35] showed that the SsPAQR1 has no mitochondrial targeting signal peptide at its N-terminal as compared to PAQR 9, 10 and 11 that have mitochondrial localization signals. MEMSAT-SVM analysis identified a signal peptide comprising the region from amino acids 1 to 39 [34]. This signal sequence possibly allows its passage through the ER to its final destination. 

A multiple sequence alignment of the derived amino acid sequence of SsPAQR1 to other fungal homologues and the human PAQR7 is included in Additional file 1. BLAST search for the predicted amino acid sequence identified this protein as 65 to 80% identical to other PAQRs of fungi such as: *Neurospora crassa, Magnaporthea oryzae, Giberella zeae,* among others. It is also shows that it is approximately 50% identical to *S. cerevisiae* Izh3 family channel protein.

### Co-immunoprecipitation (Co-IP) and western blots

**Figure 3:**
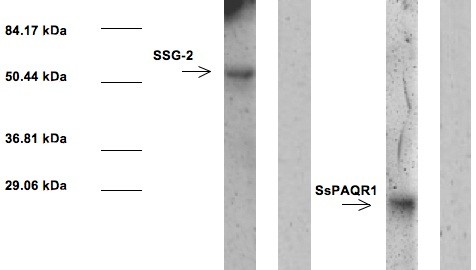
**Western Blots and co-immunoprecipitation of the SSG-2/SsPAQR1 interaction.** Whole cell free extracts of *S. cerevisiae* cells containing pGBKT7 and pGADT7 plasmids with the complete SSG-2 coding region fused to the GAL4 activation domain and cMyc, and the initial insert coding fragment identified in the yeast two-hybrid assay fused to the GAL4 DNA binding domain and HA, respectively, were co-immunoprecipitated as described in Methods. The co-precipitated proteins were separated using 10% SDS polyacrylamide electrophoresis and transferred to nitrocellulose. The nitrocellulose strips were probed with anti-cMyc antibodies (Lane 1) and anti HA antibodies (Lane 3), respectively. Lanes 2 and 4 are negative controls where no primary antibody was added. The antigen-antibody reactions were detected using the Immun-Star™ AP chemiluminescent protein detection system. Pre-stained molecular weight markers were included in outside lanes of the gel and transferred to nitrocellulose, the position of the molecular weight markers is indicated in the figure.

The SSG-2/SsPAQR1 interaction was corroborated using co-immunoprecipitation and Western Blot as shown in Figure3. Lane 1 shows the band obtained using anti-cMyc antibody that recognizes SSG-2. This band is of the expected size (58 kDa) considering that SSG-2 was expressed fused to the GAL-4 binding domain. Lane 2 shows the results obtained in the Western blot when the primary anti-cMyc antibody was not added (negative control). Lane 3 shows the band obtained using anti-HA antibody that recognizes the original SsPAQR1 fragment isolated from the yeast two-hybrid clone. This band is of the expected size (22.4 kDa) considering that only the last 38 amino acids of the protein were present and that this fragment was fused to the GAL-4 activation domain. Lane 4 shows the results obtained in the Western blot when the primary anti-HA antibody was not added (negative control).

### Yeast-based assay

**Figure 4:**
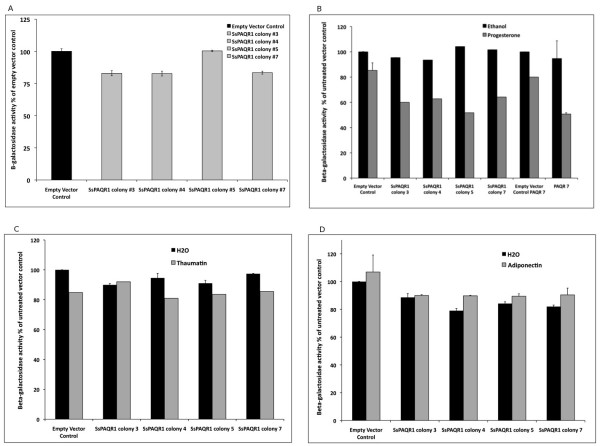
**SsPAQR1 yeast-based assay.** The agonist of SsPAQR1 was identified using a yeast-based assay as described in Methods. *S. cerevisiae* BY4742 was transformed with YEp353 (*FET3*-*lacZ*) containing a fragment of the *FET3* promoter fused to *lacZ* driven by a minimal CYC1 promoter and with pYES2CT w/wo the *sspaqr1* gene insert. *S. cerevisiae* were grown in LIM-Fe medium containing 2% galactose and *FET3* activity is measured using the *FET3-lacZ* construct as a reporter. Black bars show *FET3-lacZ* activity in yeast treated with the solvent only (H_2_O or ethanol) and gray bars show activity in yeast treated with different possible agonist; thaumatin, adiponectin or progesterone. *FET3-lacZ* activity was measured as the β-galactosidase activity expressed as the percentage of the untreated vector control. Panel (**A**) shows that SsPAQR1 does not repress *FET3-lacZ* when over-expressed in yeast by using the GAL1 promoter. Panel (**B**) shows β-galactosidase activity in cells expressing SsPAQR1 in the presence of 1 mM progesterone, panel (**C**) shows β-galactosidase activity in cells expressing SsPAQR1 in the presence of 50 μM thaumatin and panel (**D**) shows β-galactosidase activity in cells expressing SsPAQR1 in the presence of 0.1 μM adiponectin.

To identify the agonist of the SsPAQR1, a yeast-based assay was used [13]. This assay is based on the fact that PAQRs expressed in yeasts, activate a signal transduction pathway that represses the expression of the *FET3* gene. Yeast cells were co-transformed with plasmids, YEp353 (*FET3-lacZ*) and a plasmid containing the PAQR insert, either pYES2CT or pGREG536. The response of *FET3* fused to the *lacZ* gene was used as a reporter for PAQR receptor activity. Figure4A shows the effects of SsPAQR1 on *FET3-lacZ* when over-expressed in yeasts using the *GAL1* promoter for randomly selected colonies. These results show that in the absence of agonist, SsPAQR1 did not significantly repressed *FET3-lacZ* using the Student’s t-test (p>0.05)*.* Figure4B, shows that when exposed to 1 mM progesterone, transformed yeasts cells expressing SsPAQR1 elicited a significant repression of *FET3-lacZ* (Student’s t-test, p <0.05) when compared to yeast cells transformed with the empty plasmid or the SsPAQR1-containing plasmid with added ethanol (controls). A small repression of *FET3-lacZ* was observed in yeasts transformed with the empty plasmid if progesterone was added; nevertheless, the level of repression of *FET3-lacZ* was significantly larger when yeast cells transformed with the plasmid expressing SsPAQR1 were treated with the ligand (Student’s t-test, p>0.05). This figure also shows the results obtained with PAQR 7 used as a positive control. PAQR 7 is a previously characterized progesterone receptor. This figure shows the combined data of the effects of progesterone on *FET3-lacZ* expression of 4 randomly picked colonies of cells transformed with the plasmid pGREG536 containing PAQR 7. The differences on *FET3-lacZ* expression were significant using the Student’s t-test (p< 0.05). Figure4C and 4D show no significant repression of *FET3-lacZ* when thaumatin (50 μM) or adiponectin (0.1 μM) were used as ligands for the same 4 colonies transformed with the plasmid expressing SsPAQR1 when compared to the controls (Student’s t-test, p<0.05). 

### Intracellular cAMP levels in *S. schenckii* treated with progesterone

**Figure 5:**
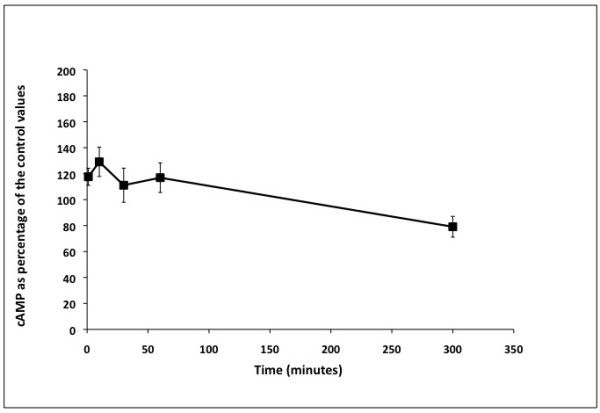
**Effects of progesterone on intracellular cAMP in*S. schenckii*.** This figure shows the cAMP response curve after the exposure of *S. schenckii* yeast cells to progesterone for different time intervals. The cells were grown in a variation of medium M for 4 days and aliquots were removed and exposed to progesterone as described in Methods. The intracellular levels of cAMP were measured as described in Methods using the cAMP Direct Immunoassay kit (Calbiochem, La Jolla, CA, USA). The cAMP concentration was determined for at least 7 independent experiments and the values expressed as percentage of the untreated controls (ethanol only) ± the standard error of the mean. Significance of the data was determined using the Student’s T test and at a p<0.05. Analysis of Variance between groups was done using Bonferroni Test for differences between means.

Figure5 shows the cAMP levels of *S. schenckii* yeast cells exposed to progesterone 0.5 mM for different time intervals (1, 10, 30, 60, and 300 minutes) before harvesting for cAMP determinations. This figure shows that there was an immediate significant increase in the levels of cAMP in cells treated with progesterone within 1 min after the addition of progesterone when compared to the controls (Student’s t-test, p>0.05). A significant decrease in cAMP levels was observed when cells were treated with progesterone for 5 h. Analysis of Variance between groups, done using Bonferroni Test for differences between means revealed that there were no differences in the cAMP levels between samples taken at 1, 10, 30 and 60 minutes following exposure to progesterone but all were significantly different when compared to that obtained after 300 min of exposure.

### Effects of progesterone on growth of *S. schenckii*

Progesterone inhibited growth of *S. schenckii* conidia in Medium M agar plates. Table1 shows the colony diameter of conidia incubated at 25°C and 35°C in medium M agar plates for 20 days at different concentrations of added progesterone. This table shows that conidia did not germinate at concentrations of progesterone of 0.05 mM or above at 35°C. These same conidia inoculated in medium M plates with different concentrations of added progesterone and incubated at 25°C grew at all concentrations of the hormone. Nevertheless the growth was significantly smaller at concentrations of progesterone 0.05 mM or above when measured as the diameter of the colony (Student’s t-test, p<0.05).

**Table 1:** **Effects of Progesterone on*S. schenckii*yeast and mycelium growth from conidia**

**Progesterone concentration (mM)**	**Average diameter of colonies incubated at 25°C (cm)**^**a,b,c**^	**Average diameter of colonies incubated at 35°C (cm)**^**a,b,c**^
0	2.40 ± 0.18	1.47 ± 0.13
0.010	2.35 ± 0.10	1.33 ± 0.11
0.050	2.10 ± 0.11*	no growth
0.125	1.78 ± 0.07*	no growth
0.250	1.47 ± 0.16*	no growth
0.500	1.22 ± 0.11*	no growth

This table shows the colony diameter attained after conidia were inoculated at 25°C and 35°C in a modification of medium M agar plates with different concentrations of added progesterone. No growth was observed at concentrations of progesterone of 0.05 mM or above, at 35°C while conidia incubated at 25°C germinated and showed growth at all concentrations of progesterone tested. The data represents the average diameter ± one std deviation of 6 independent experiments.

^a^ The cultures were incubated at the desired temperature for 20 days.

^b^ All cultures were inoculated with 5μl of a suspension containing 10^6^/μl conidia.

^c^ The values given are the average of 6 independent determinations.

* The values marked with an asterisk are significantly different from the values where no progesterone was added to the medium.

## Discussion

A seemingly universal new family of receptors, the PAQRs, that originated from ancestral bacterial hemolysin encoding genes has been described in eukaryotes [7]. Much controversy surrounds these receptors specifically, their membrane topology and the possibility of being coupled to G protein signalling pathways [17]. Nevertheless, the nature of the ligands bound by a particular receptor has been solved for most PAQRs. They have been observed to bind either the peptide hormone adiponectin or the steroid hormone progesterone [38,39]. This brings up another consideration; should the adiponectin and progesterone receptors be considered members of the same family of receptors? The fact that the nature of the ligands are so different, together with the differences observed in membrane topology between the Class I (adiponectin receptors) and Class II (progesterone receptors) suggest inherent difference between the two classes. Class I receptors have been predicted to have the N-terminal in the interior of the cell while Class II receptors have the usual GPCR topology of the N-terminal outside of the cell and the C-terminal inside the cell [8,20]. Due to the predicted membrane topology of the progesterone receptors, it is suggests that they might be a new class of GPCRs. In this paper we report a new member of the Class II PAQRs and address the issues regarding membrane topology, ligand binding and its relationship to the *S. schenckii* G alpha subunit SSG-2, in an effort to characterize the SsPAQR1.

The fact that SsPAQR1 was identified in a Y2H assay with a G protein alpha subunit as bait, offers for the first time direct evidence of the association of these receptors to the heterotrimeric G protein signalling pathways. This association was verified using Co-IP. Indirect evidence of the association of progesterone PAQRs to G proteins has been reported by other investigators. One of these instances involves fish oocyte maturation where response to a novel progesterone hormone was associated to a pertussis-sensitive Gαi subunit pathway [6,11,40].

Transmembrane analysis of the SsPAQR1 described here predicts that this protein has the 7 transmembrane domains characteristic of GPCRs like other progesterone binding members of the PAQR family. The bioinformatic analyses described above (TMHMM, SOUSI and MEMSAT-SVM) predicted that the N-terminal region is localized outside the plasma membrane while the C-terminal region is intracellular. This orientation has also been observed in progestin receptors, PAQR6 and mPRa [6]. In the case of the adiponectin members of the PAQR family such as the human adiponectin receptor 2 and 3, the orientation seems to be the opposite, as stated previously [12,41].

Bioinformatic analyses also show that SsPAQR1 and its fungal homologues from *M. oryzae*, *T. reesei*, *N. crassa* and *P. anserina*, among others belong to the PAQR receptor family. These homologues exhibit approximately 65 to 80% identity to SsPAQR1. The transmembrane domain analyses of some of these fungal homologues showed that most have the 7 transmembrane domains characteristic of the GPCRs. TMHMM analysis also shows that they have the traditional orientation of an external N-terminal domain and an internal C-terminal domain as SsPAQR1, except in the case of Izh3 where the N-terminal is inside and the C-terminal is outside (Additional file 2). It is also of interest to note that in many filamentous and dimorphic fungi where whole genomes have been sequenced, the SsPAQR1 homologues encode proteins of approximately 500 amino acids in addition to at least one more Izh homologue of smaller size (approximately 300 amino acids).

Steroid binding proteins have been described for various yeasts [42]. Many studies have predicted the existence of a progesterone receptor in the membrane of filamentous fungi such as *Rhizopus nigricans*[27-30] but the molecular basis of steroid signalling in fungi remains unresolved [43,44]. Progesterone has been reported to bind to enriched plasma membrane fractions of *R. nigricans* with high affinity and this hormone has been reported to induce an activation of G proteins that decreases in the presence of cholera toxin [29]. Nevertheless, to date no progesterone receptor has been directly identified in this or any other fungi. This work identified a membrane progesterone receptor for the first time in fungi. Progesterone was identified as the ligand corresponding to SsPAQR1 using the yeast-based assay [23,45]. This assay was used previously to identify the ligands of human PAQRs heterologously expressed in *S. cerevisae*[46]. This assay is specific for PAQRs and was intended for the study of these receptors without the intervention of other possible progesterone binding protein. Using this assay, SsPAQR1 was expressed in *S. cerevisiae* and progesterone was identified as the ligand for SsPAQR1. Yeasts carrying the empty expression vector showed that progesterone did not affect *FET3*, showing that the effect was not due to a nonspecific effect of progestrone on *S. cerevisiae.* Progesterone responsiveness was only observed if SsPAQR1 was being expressed. These results put an end to the uncertainty regarding the presence of a membrane progesterone receptor in fungi.

However, the question as to why fungi have a steroid hormone receptor remains unanswered. The effects of progesterone and other steroids on fungi have not been fully documented. In *Candida albicans* the response to steroid hormones leads to the activation of transcription of genes encoding the ATP-binding cassette of drug efflux pumps [47]. In *S. cerevisiae* exposure to progesterone results in the up-regulation of stress response genes such as those involved in transport, oxidative stress response, growth, cell division and cell wall biogenesis, among other [43].

In the filamentous fungi, most of the information regarding progesterone and fungi is related to bioconversion of the different steroid metabolites by fungi. Recently, a progesterone-hydroxylating enzyme system was studied and found to be dependent on the G protein beta subunit and cAMP in *Fusarium oxysporum*[48]. The authors proposed that progesterone is toxic to this fungus and that by the induction of the enzymes involved in the hydroxylation of progesterone, the fungus is able to reduce the toxicity associated with the hormone. This transformation results in a more soluble compound that can be excreted to the medium.

The toxicity of progesterone results in an inhibition of growth in *R. nigricans*[49]. This inhibition of growth was explained as resulting from a decrease in cAMP caused by progesterone. In this work we also report an inhibition of growth of both the mycelium and yeast forms of the fungus in the presence of progesterone, the yeast form being the most affected. Nevertheless, we could not correlate this inhibition of growth to a decrease in cAMP concentrations.

Another major area of concern regarding progesterone PAQRs is the determination of the specific signal generated upon the interaction of the receptor with its ligand. Different theories have suggested that cAMP and/or calcium could be involved. Nevertheless, even in situations where adenylate cyclase has been identified as a target of the possible effects of progesterone, there is still disagreement if the hormone causes a decrease or an increase in cAMP, and the time considered reasonable for the effect on this cyclic nucleotide to be observed [50,51]. The addition of progesterone to *S. schenckii* yeast cells prior to harvesting for cAMP determinations showed that the levels of intracellular cAMP increased during the first minute after exposure to the ligand and decreased significantly after five hours incubation with the hormone. The increase in the cytosolic concentration of cAMP could be the result of the interaction of the ligand and the receptor resulting in the activation of SSG-2 that in turn triggers the cascade of events leading to an increase in cAMP. The response to the ligand in steroid membrane receptors has been identified as occurring in 1 to 5 min in the case of sperm motility to up to 6-18 h in the case of oocyte maturation experiments [50].

The work reported here identifies the presence of a progesterone receptor in *S. schenckii* for the first time and establishes the presence of homologous of this receptor in other fungi as well. Other authors who studied the response of fungi to progesterone have proposed the existence of this receptor. Although the question still remains regarding the benefit of having such receptors in fungal cells remains open, one could argue that fungi are in contact with plant and other fungal steroids in their environment and that they have the capacity to transform these molecules to suite their needs [52].

## Conclusions

The information available concerning members of the PAQR receptor family is limited and controversial. Several investigators have proposed the existence of a progesterone receptor in fungal membranes. In this work we identified for the first time a progesterone receptor belonging to the PAQR Class II family in *S. schenckii*. A yeast-based assay similar to the one used to identify the ligand for the human PAQRs, was used to identify the ligand of this receptor. This study constitutes the first evidence of the interaction of a fungal Gα subunit with a member of the PAQR family using both yeast two-hybrid assay and co-immunoprecipitation and Western Blot. The association of a G protein alpha subunits with SsPAQR1 suggests that these receptors are G protein coupled. As with many G protein coupled receptors, the hormone affects the growth of the fungus possibly by a mechanism involving cAMP. The progesterone receptor that we have identified in *S. schenckii*, brings to a close the search for a membrane progesterone receptor in fungi.

## Methods

### Strains and culture conditions

*S. schenckii* (ATCC 58251) was used for all experiments. The yeast form of this fungus was obtained as described previously [53]. *S. cerevisiae* strains AH109 and Y187 were used for the yeast two-hybrid screening and were supplied with the MATCHMAKER Two-Hybrid System (Clontech Laboratories Inc., Palo Alto, CA). *S. cerevisiae* strain BY4742 for the yeast-based ligand-binding assay was obtained from Dr. Thomas J. Lyons, from the Foundation for Applied Molecular Evolution (Gainesville, FL).

### Nucleic acids isolation

DNA and RNA were obtained from *S. schenckii* yeast cells as described previously [54]. Poly A^+^ RNA was obtained from total RNA using the mRNA Purification Kit from Amersham Biosciences (Piscataway, NJ, USA) and used as template for cDNA synthesis.

### Yeast two-hybrid

MATCHMAKER Two-Hybrid System was used for the yeast two-hybrid assay (Clontech Laboratories Inc., Palo Alto, CA) using all 3 different reporter genes for the confirmation for truly interacting proteins as described previously [55]. For the construction of the bait plasmid, *ssg-2* cDNA was obtained from poly A^+^ RNA, transcribed and amplified by RT-PCR using the Ready-to-Go™ Beads (Amersham Biosciences) as described [55], cloned and used to transform competent *S. cerevisiae* yeast cells (Y187). Competent *S. cerevisiae* yeast cells were transformed using the YEASTMAKER™ Yeast Transformation System 2 from Clontech (BD Biosciences, Clontech Laboratories Inc.).

Poly A^+^ RNA was isolated form total RNA extracted from logarithmically growing *S. schenckii* yeast cells. Double stranded cDNA was synthesized from RNA using SMART™ Technology Kit (Clontech Laboratories Inc.). The cDNAs were amplified using Long Distance PCR and size selected using the BD CHROMA-SPIN™+TE-400 columns (Clontech Laboratories Inc.) [55].

*S. cerevisiae* yeast cells AH109 transformed with SMART ds cDNA (20μl) were selected in SD/-Leu plates, harvested and used for mating with the bait containing *S. cerevisiae* strain Y187. Mating of *S. cerevisiae* yeast cells strains Y187 (Mat-α) and AH109 (Mat-a) was done according to the manufacturer’s instructions. The expression of three reporter ADE2, HIS3 and MEL1 genes in the diploids was used as confirmation for true interacting proteins. Diploids expressing interacting proteins were selected as described previously [55]. Colony PCR was used to corroborate the presence of both plasmids in the diploid cells using the T7/3′BD sequencing primer pair for the pGBKT7/*ssg-2* plasmid and the T7/3^′^AD primer pair for the pGADT7-Rec library plasmid. PCR was used to amplify the inserts in the isolated plasmid with the T7 Sequencing Primer/3^′^AD sequencing primer pair provided with MATCHMAKER Two-Hybrid System, and the PCR product was cloned and sequenced as described previously [55].

### Co-immunoprecipitation (Co-IP)

*S. cerevisiae* diploids obtained in the yeast two-hybrid assay were grown in 125 ml flasks containing 25 ml of QDO for 16h, harvested by centrifugation and resuspended in 8 ml containing phosphate buffer saline (800μl) with phosphatase (400 μl), deacetylase (80 μl) and protease inhibitors (50μl), and PMSF (50μl). The cells were frozen in liquid nitrogen in a porcelain mortar, glass beads added and the cells broken as described previously [56]. The cell extract was centrifuged and the supernatant used for Co-IP using the Immunoprecipitation Starter Pack (GE Healthcare, Bio-Sciences AB, Bjorkgatan, Sweden) as described by the manufacturer. Briefly, 500μl of the cell extract were combined with 1-5μg of the anti-cMyc antibody (Clontech, Corp.) and incubated at 4°C for 4h, followed by the addition of protein G beads and incubated at 4°C overnight in a rotary shaker. The suspension was centrifuged and the supernatant discarded, 500μl of the wash buffer added followed by re-centrifugation. This was repeated 4 times. The pellet was resuspended in Laemmeli buffer (20μl) and heated for 5 min at 95°C, centrifuged and the supernatant used for 10% SDS PAGE at 110V/1 h.

### Western blots

Western blots were done as described by us previously [56]. The proteins were separated by electrophoresis and transferred to nitrocellulose membranes using the BioRad Trans Blot System® for 1 h at 20 volts. After transfer, the nitrocellulose strips were blocked with 3% gelatin in TTBS (20 mM Tris, 500 mM NaCl, 0.05% Tween-20, pH 7.5) at room temperature for 30-60 min. The strips were washed for 5-10 min with TTBS. The TTBS was removed and the strips incubated overnight in the antibody solution containing 20 μg of antibody anti-cMyc or anti-HA (Clontech, Corp.). Controls where the primary antibody was not added were included. The antigen-antibody reaction was detected using the Immun-Star™ AP chemiluminescent protein detection system from BioRad Corporation (Hercules, CA, USA) as described by the manufacturer.

### Sequencing of the *sspaqr1* gene

#### Rapid amplification of cDNA ends (RACE)

The 5^′^ end of the *sspaqr1* gene homologue was obtained using RLM-RACE (Applied Biosystems, Foster City, CA, USA) with *S. schenckii* cDNA as template. All RACE reactions were carried out in the ABI PCR System 2720 (Applied Biosystems). The touchdown PCR and nested PCR parameters used for the initial RACE reactions were the same as described previously [55]. Nested primers were designed to improve the original amplification reactions. Bands from the 5′ nested PCR were excised from the gel and cloned as described previously [54]. Primers for RACE were designed based on the sequence obtained from the yeast two-hybrid assay. The touchdown and nested primer used to complete the cDNA secuences of *sspaqr1* were: EFFSNAFRD-GSP (rev) 5^′^ ctggcggaaggcgttggagaagaactc 3^′^, VLGGTLFHY-NGSP (rev) 5^′^ agtagtggaacaggatgccgcccagcac 3^′^ , VFFLLFSRFF-GSPP2 (rev) 5^′^ aaaacgagaaaaaagaaggaagaaaac 3^′^, EFY-GSP (rev) 5^′^ acttggtaatgggcaggtaaaactc 3^′^, VAFYV-NGSP (rev) 5^′^ ggccagaccaacataaaacgcgacg 3^′^, MTG-GSP2 (rev) 5^′^ caccatcggcacaaagcccgtcatg 3^′^, STIW-GSP1 (rev) 5^′^ cgagttcatggtgtgccagatggtgct 3^′^, VFVA-GSP2 (rev) 5^′^ aagaagaagacggcagcaacgaacacg 3^′^, YGE-GSP (rev) 5^′^ tcgagtcgatggagacctcgccata 3^′^, FDL-GSP2 (rev) 5^′^ ccagttcggtcaagaacaagtcaaa 3^′^.

The complete cDNA coding sequence of the *sspaqr1* gene was obtained using reverse transcriptase polymerase chain reaction (RTPCR). For RTPCR, RNA was extracted as described previously [54]. The cDNA was obtained using the RETROscript^™^ First Strand Synthesis kit (Ambion, Applied Biosystems, Foster City, CA, USA) and used as template. : VLCLAYD(fw)/GGCDWYL(rev) primer pair. The sequence of these primers were the following: 5′ tatttgtgtctttcttac 3′ and 5′ ataccattaacaacagcc 3′, respectively. The following PCR parameters were used: an initial denaturation step at 94°C for 30 sec, followed by 25 cycles of denaturation at 94°C for 5 sec, annealing at 40°C for 10 sec, and extension at 72°C for 2 min. The RTPCR products were cloned as described previously [54] and the inserts sequenced using commercial sequencing services from Davis Sequencing (Davis, CA, USA).

### Bioinformatics sequence analysis

The theoretical molecular weight of SsPAQR1 was calculated using the on-line ExPASy tool (http://expasy.org/tools/pi_tool.html). The protein classification was performed using the PANTHER Gene and Protein Classification System (http://www.PANTHERdb.org) [31]. On-line database search was performed with the BLAST algorithm (http://www.ncbi.nlm.nih.gov/BLAST/) with a cutoff of 10^-7^, a low complexity filter and the BLOSUM 62 matrix [57]. Transmembrane domains were identified using TMHMM Server v. 2.0 (http://www.cbs.dtu.dk/services/TMHMM) [32] and visualized with TOPO2 (http://www.sacs.ucsf.edu/TOPO2/). SOSUI server (http://bp.nuap.nagoya-u.ac.jp/sosui/sosuiframe0E.html) and PSIPRED Protein Prediction server, MEMSAT-SVM (http://bioinf.cs.ucl.ac.uk/psipred/) were also used to identify transmembrane domains [33,34,58]. Cellular localization of the SsPAQR1 was done using PSORT II Server (http://PSORT.ims.u-tokyo.ac.jp/) [35] and for the identification of mitochondrial signal sequence Predotar (http://urgi.versailles.inra.fr/predotar/predotar.html) [36], TargetP 1.1 server (http://www.cbs.dtu.dk/services/TargetP) [37] and MitoProt (http://ihg.gsf.de/ihg/mitoprot.html) [59] servers were used. Multiple sequence alignments were built using MCOFFEE (http://igs-server-cnrs-mrs.fr/tcoffee/tcoffee_ cgi/index.cgi) [60]. The alignment in Additional file 1 was visualized using GeneDoc (http://www.psc.edu/ biomed/genedoc). The accession numbers of the sequences used for the multiple sequence alignment of G protein subunits were: *S. schenckii*, ACA43006.1; *M. oryzae*, XP_362234.1; *Trichoderma reesei*, EGR51560.1; *N. crassa*, XP_965338.1; *Chaetomium globosum*, XP_001221101.1; *F. oxysporum*, EGU81989.1; *Podospora anserina*, XP_001912493.1; *Gibberella zeae,* XP_381240.1; *Paracoccidioides brasiliensis*, EEH45107.1; *Aspergillus nidulans*, EAA62332.1; *S. cerevisiae*, (Izh3p), NP_013123.1 and *Ajellomyces capsulatus*, EER42609.1.

### Yeast-based assay

*S. cerevisiae* strain BY4742 cells (MATα his3Δ1 leu2Δ0 lys2Δ0 ura3Δ0) co-transformed with plasmids, YEp353 (*FET3-lacZ*) and pYES2CT (1μg each) with the S.c. EasyComp^™^ Transformation Kit (Invitrogen Corp. Carlsbad, CA, USA) was used for the ligand-binding assay. YEp353 (*FET3-lacZ*) contains a fragment of the *FET3* promoter that includes the iron response element fused to *lacZ* driven by a minimal CYC1 promoter. The complete coding sequence of *sspaqr1* gene was cloned into pYES2CT allowing galactose-inducible SsPAQR1 expression via GAL1 promoter. The YEp353 (*FET3-lacZ*) and pGREG536 w/wo the PAQR7 insert were generously provided by Dr. Thomas J. Lyons from the Foundation for Applied Molecular Evolution. Transformants were selected in SD (-leu/-ura). For the receptor activity assay, the transformants were grown overnight in synthetic defined (SD) media without the appropriate amino acids (OD_600_, 1-1.5). The overnight culture was used to inoculate 5 ml of LIM-Gal medium (low iron media, LIM-FE, with 2% galactose as carbon source) to induce full expression of the PAQR gene driven by the GAL1 promoter and incubated at 30°C with shaking. Five hundred μl of the cells were added to 4.5 ml LIM-GAL medium with the added ligand (50.0 μM thaumatin; 0.1μM adiponectin; 1.0 mM progesterone) (Sigma-Aldrich, St. Louis, MO, USA and Phoenix Pharmaceuticals, Phoenix, AZ, USA) or the solvent alone (controls) and incubated overnight at 30°C with shaking. The cells were centrifuged and resuspended in 250 μl of breaking buffer, OD_600_ of the suspension was determined and glass beads were added together with 12.5 μl of PMSF. The cells were vortexed at least 6 times with chilling period in between vortexing periods. More breaking buffer was added at the end (250μl), mixing well and the extract recovered. Ten μl of this extract were added to 990 μl of Z buffer (60 mM NaH_2_PO_4_, 40 mM Na_2_HPO_4_, 10mM KCl, 1 mM MgSO4, pH 7.0) and the mixture incubated at 28°C for 5 min. The reaction was initiated by adding 200 μl of a stock solution of ONPG (4 mg/ml) and the mixture incubated for 10 min at 28°C. The reaction was terminated by adding 500 μl of 1 mM Na_2_CO_3_ and the optical density recorded at 420 nm. For all experiment, equal volumes of the appropriate solvent were added to untreated cells as control for vehicle effects. The data shows the individual results obtained with 4 different colonies transformed with the above-mentioned plasmids. The data for PAQR 7 represents the combined data of 4 different colonies.

### Cyclic 3^′^, 5^′^-adenosine monophosphate assay (cAMP)

*S. schenckii* yeast cells were grown from conidia for 4 days at 35°C as described previously [53]. Ten μl of ethanol or progesterone (0.5 mM) were added to 1 ml aliquots (10^6^ cells/ml) of the culture for a predetermined period of time (1, 10, 30, 60 and 300 min). The cells were centrifuged and 0.01 mM HCl (400 μl) was added to the cells together with glass beads. The cells were vortexed for 1 min and frozen at -80°C 3 times, followed by centrifugation. One hundred μl of this suspension was assayed colorimetrically for cAMP using the cAMP Direct Immunoassay kit (Calbiochem, La Jolla, CA, USA). The cAMP concentration was determined for at least 7 independent experiments and the values expressed as percentage of the untreated controls (ethanol only).

### Effects of progesterone on growth of *S. schenckii*

Conidia were obtained from 5 day old mycelial slants growing in Saboureau dextrose agar by gentle re-suspension with sterile distilled water. Cultures were inoculated in medium M agar plates with 5 μl of a suspension containing 10^6^/μl conidia. Different concentrations of progesterone, ranging from 0.00 to 0.5mM were added to the medium. Cultures were incubated at the desired temperature (25°C or 35°C) for 20 days. The diameter of the colonies was measured at the end of this time period. The values given are the average of 6 independent determinations ± a standard deviation.

### Statistical analysis

Data was analysed using Student’s t-test. A p-value of less than 0.05 was used to determine statistical significance. For the time series of the cAMP assay, an analysis of variance with repeated measures using a post-hoc Bonferroni test was used to determine statistical significance.

## Competing interests

All of the authors state that they have not received any fees, funding or salary, nor hold stocks from any organization that in any way will gain or loose financially from the publication of this paper. No authors are at the present applying for any patent related to the content of this paper.

## Authors’ contributions

WGV did all the studies described in this manuscript including the yeast two-hybrid assay that identified SsPAQR1 as a SSG-2 interacting protein. She also did the Co-IP experiments, ligand assays, cAMP determinations and the sequencing of the SsPAQR1. This work was done as part of her research for the PhD degree. RGM participated and supervised the bioinformatic study of the proteins and statistical analysis calculations. NRV designed the study, drafted the manuscript, participated in sequence alignments, data and statistical calculations, and domain characterizations. All authors read and approved the final manuscript.

## Supplementary Material

Additional file 1 **Amino acid sequence alignments of SsPAQR1 to other fungal protein homologues.** The predicted amino acid sequence of *S. schenckii* SsPAQR1 and other fungal homologues proteins were aligned using MCoffee. In the alignment, black shading with white letters indicates 100% identity, gray shading with white letters indicates 75-99% identity; gray shading with black letters indicates 50-74% identity. Blue lines indicate the transmembrane domains of the SsPAQR1. Click here for file

Additional file 2 **TMHMM analysis of SsPAQR1 fungal protein homologues.** The TMHMM analysis was done using sequences retrieved from GenBank by means of BLAST. Sequences A to J correspond to: *A. capsulatus*, *A. nidulans*, *C. globosum*, *F. oxysporum*, *G. zeae*, *M. oryzae*, *N. crassa*, *P. anserina*, *P. brasiliensis* and *S. cerevisiae* (Izh3), respectively. Click here for file
