# Supplementary material for: Characterization and ligand identification of a membrane progesterone receptor in fungi: existence of a novel PAQR in Sporothrix schenckii
Source: BMC Microbiol. 2012 Sep 7;12:194. doi: 10.1186/1471-2180-12-194 (PMC3488014; doi:10.1186/1471-2180-12-194)
Supplement: Additional file 1 — Amino acid sequence alignments of SsPAQR1 to other fungal protein homologues. The predicted amino acid sequence of S. schenckii SsPAQR1 and other fungal homologues proteins were aligned using MCoffee. In the alignment, black shading with white letters indicates 100% identity, gray shading with white letters indicates 75-99% identity; gray shading with black letters indicates 50-74% identity. Blue lines indicate the transmembrane domains of the SsPAQR1. [file 1471-2180-12-194-S1.pdf]

```
S.schencki    1 : MDFAASKMSCN----ATASGRGPAEGTN-EAKTSGT-EQITADV--APVRRRQRSSFSRPRRKSLANH-IIDSEESLLLKFDLFLTELERRLESLESYGE
M.oryzae      1 : MALSPSELCTSQLNRASPSPISANEDDY-SSAQASG-REGSDDV--RPRRRRHSSYFPQRRKSIVNH-IMDGEEHILLKVDLFLTELERRLDFLESYGE
T.reesei      1 : MAGVT--TA-----LTSACGFSHDSDAA-SSTATSG-SDPRREA---QQARRRRHSFFIPRRKSIVGH-IMEGEENLLLKVDLFLTELERRLEFIENYVD
F.oxysporu    1 : MSDVT--TA-----ITTACAYKAR-DGDNIASTSG--SDIRPDV---E-PRRRRHSFFIPRRRSIVGH-IMDGEEGLLLKVDLFLSELERRLDFIENYVD
G.zeae        1 : MSDVT--TA-----ITTACTYKAR-DSDNIATTSG--SDIRPDA---G-PRKRRHSFFIPRRRSIVGH-IMDGEEGLLLKVDLFLSELERRLDFIENYVD
N.crassa      1 : -------MECA-----SPFSFAAHDDNT-EARASGA-ANAP--E--RPALRNRRKSQLNARRKSFVNS-IVDGEEALLLKMDAFLVELESRLEYWENYGE
C.globosum    1 : MDVSS-NMGCP-----APFSFSSCDDAA-KG-ASSG-AEPH--N--RPAAR-RRHSHFMQRRKSIVNH-LMDGEEALLLKLDLFLTELERRLEYWENYAE
P.anserina    1 : MDVSP-GLGCP-----SPFTFSACEDAP-EAGQSSG-AQAQ--E--RPALRQRRPSHFKQRRKSLVNQ-FMEGEEGLLLKLDLFLTNLEKKLEDWESYGE
P.brasilie    1 : MPCT-----------GPSVTLESYADSKQSSNASGL-AVPDERA-NLLRNTRRRHSFHTSRRLSC-DY----DADAIFLRVELFLTEMERRLQWLEEYRQ
A.capsulat    1 : MACT-----------APSVTLESYAYLDPASNASGL-ATPDERS-GFIRNRRRRHSFHTNRRLSC-DH----DADAIFLRVELFLTEMERRLQWLEEYRQ
S.c.IZH3      1 : MMDSSSKSLTQ----YIP---SPMGSLSR-LKQKGVDNFQKVKKSGKS-IYNYNYSKFVPHPFSTIDESVKHSESGRYDDLEIIRPTKEKEVTS------
A.nidulans    1 : MACS-----------TGSVTIESYLDTAVSS------ATPSEET-ALLRDRKRRHSFHTARKLSC-DY----DADAIFLRVELFLAELERRLHWIEQYRK

S.schencki   92 : ---VSIDSSIARAYATLQVVRTRCSQASEEVIGAGRRRLQIMVETIETRYQEALVAAESLNEKARVSIELLDDMLSDFENHAHKFRERSISHATD-----
M.oryzae     96 : ---ISLDASIARAYNTLQAVRTGCSHVSEEVIGAGRRRVHIMVETLEARYQDALAAADSLHEKACVGIDLLDTMLSDFEDHASKFRERSLANAAD-----
T.reesei     88 : ---LSKDSSISRAFSTLQAVRTRCSQASEEVIGAGRRRLHIMVETLETRYQETLEATETLHDKAVLGIELLEGMLSEFESRAYKLREQGFANAAT-----
F.oxysporu   86 : ---LSKDSSISRTFSTLQAVRSRCSHASEEVLGAGRRRLHIMVDTLEARYKETLEAAESLNEKAHVGVDLLENMLSDFETRAYKLREQGFANAAN-----
G.zeae       86 : ---LSKDSSISRTFSTLQAVRSRCSHASEEVLGAGRRRLHIMVDTLETRYKETLEAAESLNEKAHMGVDLLETMLSDFETRAYKLREQGFANAAN-----
N.crassa     82 : ---LTLDSSIEVALSTLQAVRTRCSHVSEEVMGAGRRRLHVLVETLETSYHEAMAAAGSLNEKAKVGIDLLDGLLEDLETQAAKLREKGLANAAE-----
C.globosum   86 : ---LSLDSNISAALETLQAVRTRCSQASEEVMGAGRRRLHVMVETLESSYHEALSAASSLNEKAKVGIELLDEMLQEMEEQVAKFREKGLANAAE-----
P.anserina   88 : ---LSLDSGISAAYSTLQAVQERCSQVSEEMMGAGRRKLHVMVETLESGYQDAMAAAESLNEKAKIGIEVLDDLLEDMENQAAKFRERGLANAAE-----
P.brasilie   83 : SHMTHIDARLKRGYAALLSVRDSCCYASGELMGSGKRRAKILVETLEDRYNEVLATKETFEQKAHASMRLMENFLGELELAAQSVRDRGLYSTLDDGW--
A.capsulat   83 : SHMTHIDSRLRRGYAALVSVRDSCSHASGELMGGGKRRAKILVETLEERYKEVLATKETLEQKAQASMRLMENFLGELEAGAQAVRDRGLYGTLDDGW--
S.c.IZH3     86 : ---------SVYKRNSG-------KSLNTESQFSLGDSDAATLVNSVATFKLNNASTSTSLVSSSSTVCSQAKSSLRSPTSRLNDTKIKEENNYISSVKDY
A.nidulans   78 : SHMVQIDTSLRRAYATLEAVRDSCSHASGELMGSGKKRAKILVETLESRYNDALATKETLEQKAQAGVRLMESFLTELESRADAVRDRGVYGALDDGW--

S.schencki  184 : --AAGLFMGESRRVMDEGIGRAREVVDESFERAKKAAETLEEHIQRAIANSRKNGLLSYEELPVPWRINPHIIRGYRFSETKAACFRS-MFRL---SNEL
M.oryzae    188 : --AAGAFMGEGRRVVDEGIGRAREVVDEGFAAAKWAAESLEDHIQRAVARAGKHGLIRYEDLPMPWRTNPHILKGYRFSETKLACIKSGMFGI---SNEL
T.reesei    180 : --AAEAFMDEGRRVANESIERAKSVVDEGIERAVRAALSLEEHIQQAVLLARDRGLLLYDELPTPWRNNPHILKGYRFRETKIECITS-VFNM---SNEF
F.oxysporu  178 : --AAEAFMDEGRRVANEGIERA------------IQAALSLEEHIQQAIVLAKEGRLLSYDDLPSPWRNNPHIHKGYRFTESKLECVRS-AFNL---SNEL
G.zeae      178 : --AAEAFMDEGRRVANEGIERA------------IQAAYSLEEHIQQAIVLAKEGRLISYDDLPSPWRNNPHIHKGYRFTESKLECIRS-VFTP---SNEL
N.crassa    174 : --S---LMGEGKRVMEEGIERAMR------------AAENLEDHIQRAINQAREKGLISYEELPIPWRINPHIQKGYRFSENKLACIRS-AFTF---SNEL
C.globosum  178 : --S---LMDEAHRVVDGGIERAIR------------AAETLEDHVQRAIARAREHGLLRYEDLPMPWRTNPHITKGYRFSETKLACVRS-AFSF---SNEL
P.anserina  180 : --V---LMDEAHRVVDGGIERAMR------------AAESLEDHVQRAIARAREHGLLHYDDLPVPWRINPHIKKGYRFSETKLACVRS-AFGF---SNEL
P.brasilie  181 : -RAVEPSLTHAREVMDESLAHAREVMDESIEHAR---EALQKRIERAIQLAKEQRLIHYSDLPHPWRVNPHILQGYRFTTSKIECVTS-VFTF---SNEL
A.capsulat  181 : -RAVEPGLTH------------AREVMDESVERAR---EALQKSIERAIQLANEKRLIQYSDLPHPWRVNPHILQGYRFTTSKIECVTS-VFTF---SNEL
S.c.IZH3    171 : CGPMRKSMVKTEILIEEPLNP------------TTDIKSFINSYNHGKAYSLGETQHLHYYQLPFPWRENRYIIHGYRFYNTHSKSLLS-IFNWYGWHNET
A.nidulans  176 : -KAVDSTLVQ------------AREVMDEGIERARQVKDALRENIDHAIMLAKEKRLISYSDLPAPWRINPHILSGYRFHSSKVECLTS-VFTF---SNEL
```

```
S.schencki 278 : VNIWSHGLGIVVVLSIALYFYPTSVNFHLSTKTDV--FIAAVFFFAACQCLVCSTIWHTMNSIADADLISSLACVDYTGISLLVAASIMTTEYTAFYCEP
M.oryzae   283 : VNIWSHALGLVLVLAVAFYFYPTSPNFSLSTKTDV--FIAAVFFFAACQCLVCSTIWHTMNSIADAHLISSLACVDYTGISMLIAASIMTTEYTAFYCDP
T.reesei   274 : INIWSHALGLILVLAVALYFYPSSANFYLSTKTDV--VVAAIFFMMACLTLVCSTIWHTMNAVADANAVSMFACVDYTGISLLIAASIITTEYTAFYCDP
F.oxysporu 261 : VNIWSHALGLILVLAIALYFYPNTANFTLSTKSDV--FVAGVFFVMACLTLVCSTVWHTMNAVADVDAISIFACVDYTGISLLIAASIMTTEYTAFYCDP
G.zeae     261 : FNIWSHALGLVLVLAIALYFYPNTVNFTLSSKSDV--FVAGVFFVMACLTLVCSTIWHTMNAVADVDAISIFACVDYTGISLLIAASIMTTEYTAFYCDP
N.crassa   254 : INIWSHAIGLILVLAVAFYFYPTSINFSQSTTTDI--VIAAIFFFAACQCLACSVIWHTMNSVADVNLISMFACVDYTGISLLIAASIITTEYTAFYCEP
C.globosum 258 : VNIWSHTIGLVLVLAVAFYFYPTSTNFSQSSKADI--FIAAVFFFAACQCLICSVIWHTMSSVADVGLISMFACVDYTGISLLIAASIMTTEYTAFYCEP
P.anserina 260 : VNIWSHAIGLVLVLAVAFYFYPTSTNFSLSSKADI--FVAAVFFFAACQCLVCSTIWHTMNSVADVDLISMFACVDYTGISLLIASSIMTTEYTAFYCDP
P.brasilie 273 : FNIWSHFIGLIIVLAIAFYFYPSNPNFSASTTTDV--AIAGIFFLAACKCLVCSTLWHTMNGIASQPLMERFACVDYTGISLLVAASIVTTEYTAFYCEP
A.capsulat 262 : FNIWSHLIGLLIVLAVAFYFYPSNPNFSLSTKTDI--AIAAIFFIAACKCLVCSTLWHTMNSIASQPLMERFACVDYTGISLLVAASIVTTEYTAFYCEP
S.c.IZH3   259 : SNIWSHLLGAIYIIYLAIYDFPQSEVWRNSQVPPQARWIVFMFLAAALKCMLSSVFWHTFNGTSFLKLRSKFACVDYSGITILITASILTTEFVTMYSCY
A.nidulans 260 : VNIWSHLIGLIIVLSIAFYFYPLNPNFHLSTNSDT--LVAAVFFFAACKCLVCSTLWHTMNSIADQPLMERFACVDYTGISLLVAASIVTTEYTAFYCEP

S.schencki 376 : FSRWVYISMTAVLGIGGVMLPWHPFFNRADMAWARVAFYVGLAMTGFVPMVQIMTTRGMSFVWEFYLPIT-KSLLVYLTGAMVYASKVPERWRPG-----
M.oryzae   381 : VSRWIYMSLTAILGIGGVILPWHPRFNGADMAWARVAFYVSLGATGFLPILQLSLTRGADYVYEFYTPIA-ESIAVYVFGALIYASKIPERWYPG-----
T.reesei   372 : ISRWTYMCLTAMLGIGGVILPWHPKFNGADMAWARVGFFVGLALTGFMPILQLYFSHGPEFVYNFYSPIS-KSLLVYLSGAIVYASKVPERWFPG-----
F.oxysporu 359 : VSRYVYMGLTAFLGIGGVILPWHPRFNGADMAWARVAFFVGLALTGFMPMVQLGWTHGLDFVYNFYSPIS-KSMLVYFTGAVVYASKIPERWFPG-----
G.zeae     359 : VSRYIYMGLTAFLGIGGVILPWHPRFNGADMAWVRVAFYVGLALTGFLPMVQLGWTHGLDFVYDFYSPIS-KSMLVYLSGAFVYASKIPERWYPG-----
N.crassa   352 : VSRWVYMIATAFLGVGGVILPWHPRFNGQDMAWVRVAFYIGLSASGFLPIFQIWLTRGGMSVWEHYSPIL-ESLFVYFLGALVYASKVPERWCPG-----
C.globosum 356 : VSRWVYMVTTALLGIGGVVLPWHPRFNGADMAWARVAFYVALAATGFLPIVQLSIARSPEAVFEFYTPIG-KSLFVYLLGALVYASKVPERWRPG-----
P.anserina 358 : VSRYAYMITTALLGVGGVILPWHPKFNGADMAWARVAFYCGLGATGFLPILQISLTRSFASAMEFYGPIG-KSIGVYLLGAIVYASKVPERWCPG-----
P.brasilie 371 : VSRWIYIVTTSTLGIAGVILPWHPTFNRSDMAWARVAFYVTLAATGFAPIAQLSLTRSLGWSLYFYAPLL-KSLGVYLLGALVYASQIPERWHPG-----
A.capsulat 360 : TSRWIYIITTSTLGIAGVILPWHPTFNRADMAWARVAFYVTLAATGFAPIAQLSLTRSLGWCLYFYAPLL-KSLGVYLLGAMIYAMQIPERWLPG-----
S.c.IZH3   359 : WAMYTYMSISLALGVFGVFMNWSPRFDRPEARPLRIRFFILLATMGVLSFLHLIFLTDLHYAATLFSPVTYKSVVWYLVGVVFYGSFIPERFRSDVQVDK
A.nidulans 358 : TSRWVYILLTMSLGIGGVILPWHPTFNRADWAWVRVAFYVTLALTGFAPLAQLTYARGFSWCLYFYAPVM-KSILVYFVGACVYASQIPERWKPG-----

S.schencki 470 : ---------------------------------------------MFDYIGGSHNLWHLAVLGGILFHYSAMQEFFSNAFRQAEDGCPA--Y
M.oryzae   475 : ---------------------------------------------CFDYFGGSHNLWHLAVLGGIVFHYIAMQEFFSTAFKHAEAGCGA--Y
T.reesei   466 : ---------------------------------------------CFDYIGGSHNLWHAAVLGGILFHYTAMQEFFANAFSRAEGGCPA--Y
F.oxysporu 453 : ---------------------------------------------CFDYVGGSHNLWHAAVLGGILFHYSAMQTFFANAFHRAEAGCPS--Y
G.zeae     453 : ---------------------------------------------CFDYIGGSHNLWHAAVLGGILFHYSAMQAFFANAFHRAEGGCPS--Y
N.crassa   446 : ---------------------------------------------MFDYVGGSHNLWHMAVLGGILFHYNAMQEFFSNAFRRAQDACPM--Y
C.globosum 450 : ---------------------------------------------MFDYVGGSHNLWHIAVLGGILFHYTAMQELFAHAFKLARDGCTA--Y
P.anserina 452 : ---------------------------------------------MFDYCGGSHNLWHIAVLGGILFHYKAMQAFFSHAFALAQDGCAV--Y
P.brasilie 465 : ---------------------------------------------FFDYLGGSHNIWHVAVLGGILFHYGAMQDLFTGAFVRAEGECPTLVV
A.capsulat 454 : ---------------------------------------------FFDYLGGSHNIWHVAVLGGILFHYGAMQDLFTGAFVRAEGECPSLTM
S.c.IZH3   459 : TIPTNYELSTDLEIITKQREIHFREVPTAHSKCSSCPSHAKSFKSLWWVDYFGCSHTFWHFFVVLGVIGHYRAILDMFAKRWILS---------
A.nidulans 452 : ---------------------------------------------LFDYIGGSHNIWHLAVLGGILFHYLAMQDLFANAFQRAKGECPNLTS
```
