# Supplementary figures and images for: Characterization and ligand identification of a membrane progesterone receptor in fungi: existence of a novel PAQR in Sporothrix schenckii
Source: BMC Microbiol. 2012 Sep 7;12:194. doi: 10.1186/1471-2180-12-194 (PMC3488014; doi:10.1186/1471-2180-12-194)

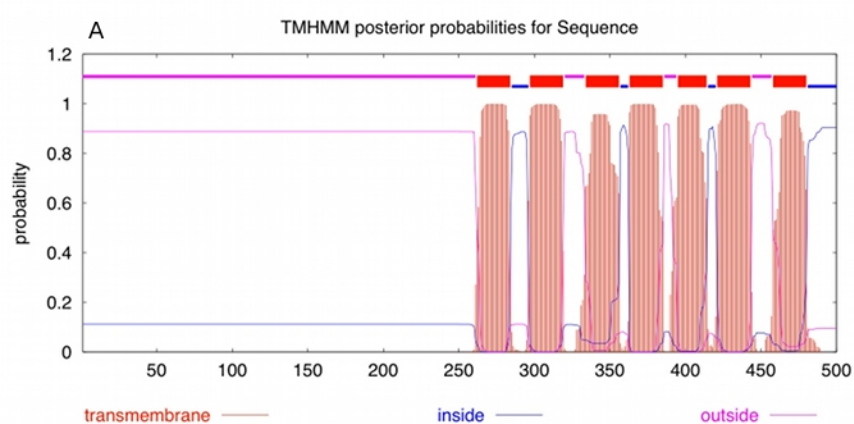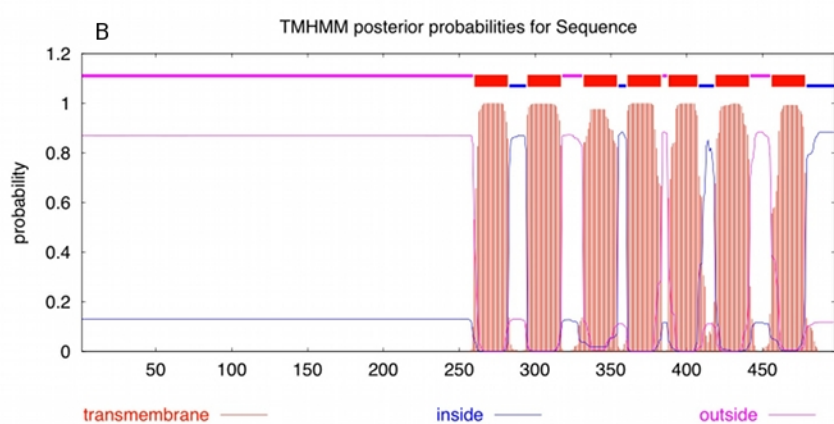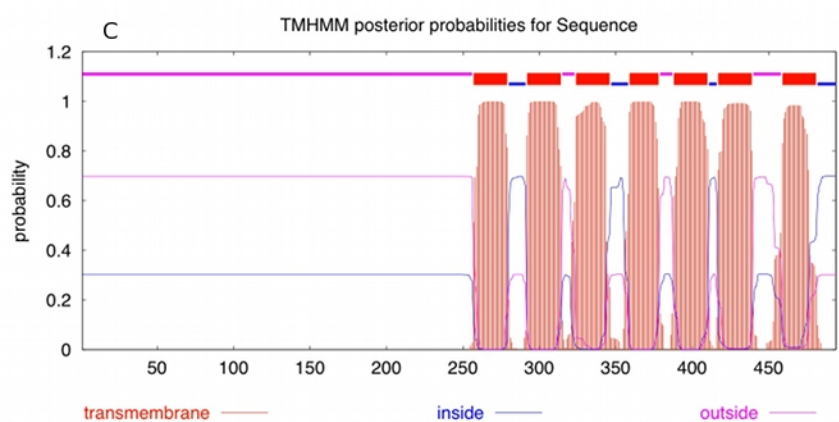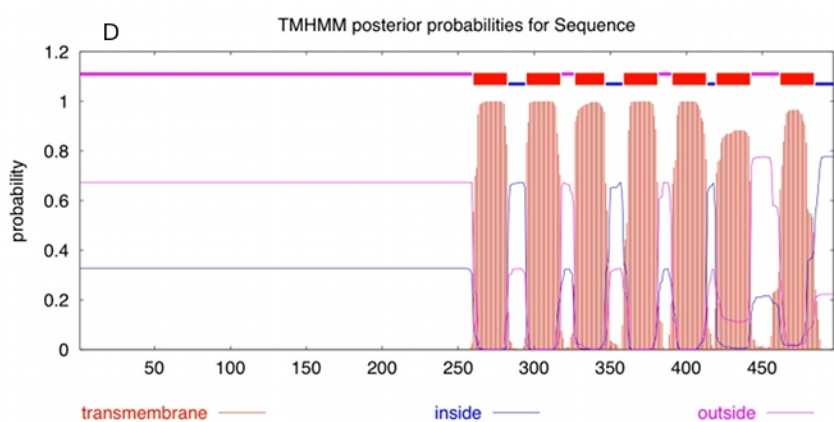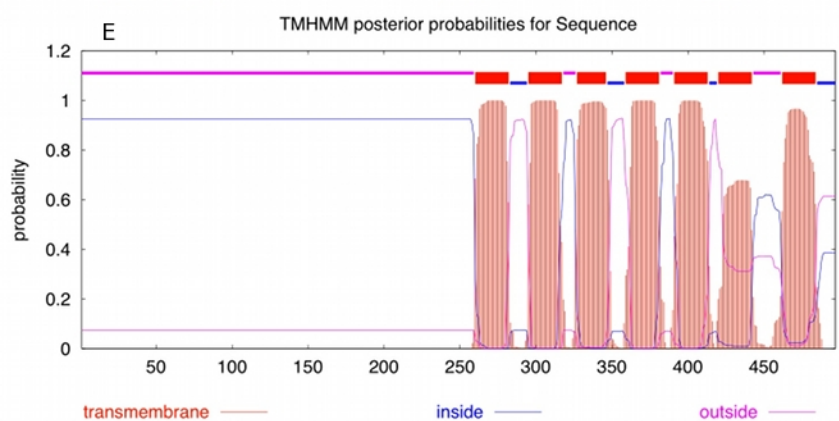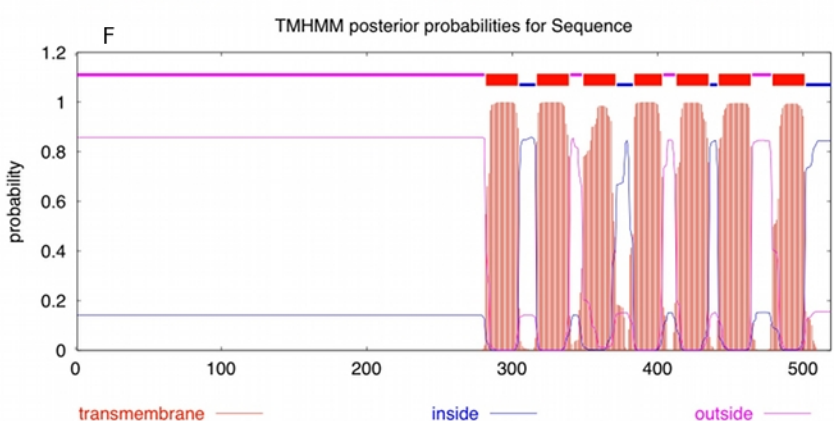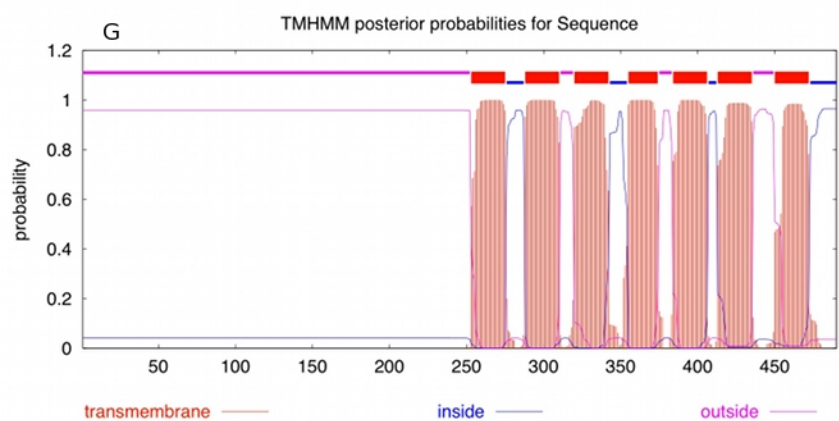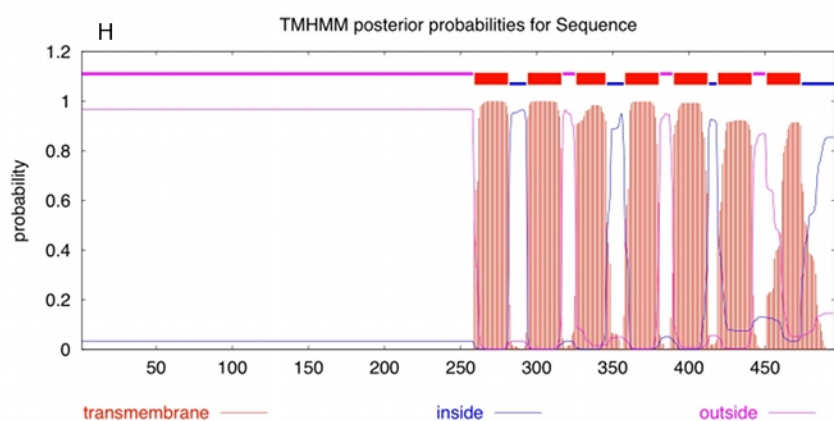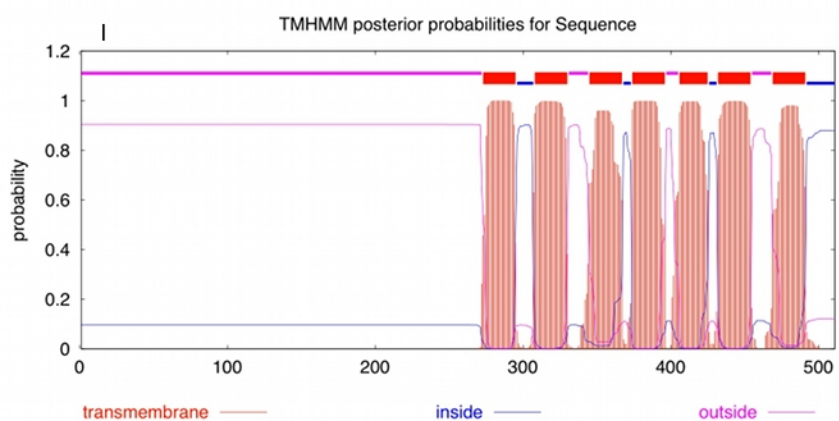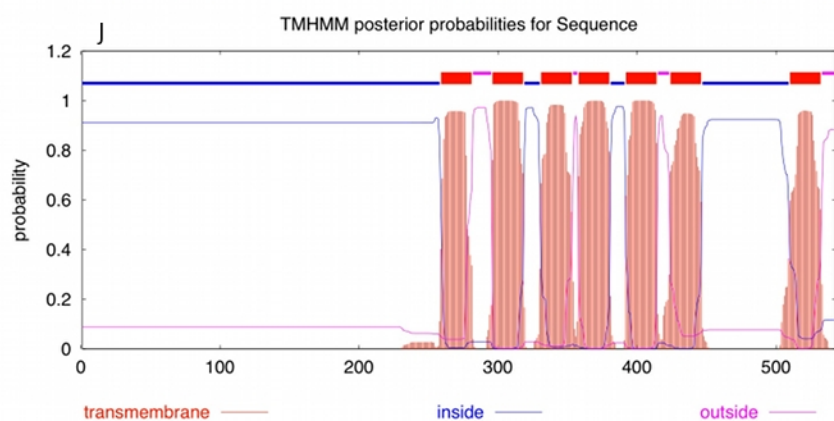

Supplement: Additional file 2 — TMHMM analysis of SsPAQR1 fungal protein homologues. The TMHMM analysis was done using sequences retrieved from GenBank by means of BLAST. Sequences A to J correspond to: A. capsulatus, A. nidulans, C. globosum, F. oxysporum, G. zeae, M. oryzae, N. crassa, P. anserina, P. brasiliensis and S. cerevisiae (Izh3), respectively. [file 1471-2180-12-194-S2.pdf]
